# Nanolitre real-time PCR detection of bacterial, parasitic, and viral agents from patients with diarrhoea in Nunavut, Canada

**DOI:** 10.3402/ijch.v72i0.19903

**Published:** 2013-04-08

**Authors:** David M. Goldfarb, Brent Dixon, Ioana Moldovan, Nicholas Barrowman, Kirsten Mattison, Chad Zentner, Maureen Baikie, Sabah Bidawid, Francis Chan, Robert Slinger

**Affiliations:** 1 Department of Pediatrics, McMaster University, Hamilton, ON, Canada; 2 Qikiqtani General Hospital, Iqaluit, NU, Canada; 3 Food Directorate, Bureau of Microbial Hazards, Health Canada, Ottawa, ON, Canada; 4 Department of Pathology and Laboratory Medicine, Children's Hospital of Eastern Ontario, University of Ottawa, Ottawa, ON, Canada; 5 Department of Pediatrics, CHEO Research Institute, University of Ottawa, Ottawa, ON, Canada; 6 Department of Health and Social Services (Nunavut), Iqaluit, NU, Canada

**Keywords:** multiplex real-time PCR, detection, pathogens, *Campylobacter* spp., *Cryptosporidium* spp., *Clostridium difficile*, viral agents, diarrhoea, gastroenteritis, Nunavut, Arctic

## Abstract

**Background:**

Little is known about the microbiology of diarrhoeal disease in Canada's Arctic regions. There are a number of limitations of conventional microbiology testing techniques for diarrhoeal pathogens, and these may be further compromised in the Arctic, given the often long distances for specimen transport.

**Objective:**

To develop a novel multiple-target nanolitre real-time reverse transcriptase (RT)-PCR platform to simultaneously test diarrhoeal specimens collected from residents of the Qikiqtani (Baffin Island) Region of Nunavut, Canada, for a wide range of bacterial, parasitic and viral agents.

**Study design/methods:**

Diarrhoeal stool samples submitted for bacterial culture to Qikiqtani General Hospital in Nunavut over an 18-month period were tested with a multiple-target nanolitre real-time PCR panel for major diarrhoeal pathogens including 8 bacterial, 6 viral and 2 parasitic targets.

**Results:**

Among 86 stool specimens tested by PCR, a total of 50 pathogens were detected with 1 or more pathogens found in 40 (46.5%) stool specimens. The organisms detected comprised 17 *Cryptosporidium* spp., 5 *Clostridium difficile* with toxin B, 6 *Campylobacter* spp., 6 *Salmonella* spp., 4 astroviruses, 3 noroviruses, 1 rotavirus, 1 *Shigella* spp. and 1 *Giardia* spp. The frequency of detection by PCR and bacterial culture was similar for *Salmonella* spp., but discrepant for *Campylobacter* spp., as *Campylobacter* was detected by culture from only 1/86 specimens. Similarly, *Cryptosporidium* spp. was detected in multiple samples by PCR but was not detected by microscopy or enzyme immunoassay.

**Conclusions:**

*Cryptosporidium* spp., *Campylobacter* spp. and *Clostridium difficile* may be relatively common but possibly under-recognised pathogens in this region. Further study is needed to determine the regional epidemiology and clinical significance of these organisms. This method appears to be a useful tool for gastrointestinal pathogen research and may also be helpful for clinical diagnostics and outbreak investigation in remote regions where the yield of routine testing may be compromised.

Diarrhoeal diseases continue to cause significant morbidity even in developed countries like Canada, and in some cases they lead to severe complications. For example, infections with exotoxin-producing *Escherichia coli* and *Campylobacter* spp. may cause renal failure and a type of paralysis known as Guillane-Barre Syndrome, respectively, and severe *Clostridium difficile* infections can result in death. Viral gastroenteritis caused by norovirus is generally of short duration, but can infect a high proportion of the population at the same time, which can lead to closures of schools and hospitals. Parasitic infections are marked by prolonged durations of gastrointestinal symptoms and can contribute to nutrient deficiencies and malnutrition.

People living in northern Canadian communities may be at particular risk for infectious diarrhoea. Potentially contaminated water supplies, consumption of traditional foods (country foods) prepared from animals carrying infectious agents (1), and overcrowded housing, leading to person-to-person spread of infectious agents, are all potential factors that may increase the risk of gastrointestinal infections.

Only a small number of studies of diarrhoeal infections in residents of northern North America have been performed. In a U.S. study of diarrhoea-associated hospitalisations and outpatient visits among American Indian (AI) and Alaska Native (AN) children, the rate of diarrhoea-associated hospitalisation among AI/AN infants was nearly twice the rate among infants from the general U.S. population (262.6 and 154.7 of 10,000, respectively). The rate of diarrhoea-associated outpatient visits among AI/AN children was also higher than for children from the general U.S. population (2,255.4 vs. 1,647.9 of 10,000, respectively), again indicating a higher burden of disease in the AI/AN children (2). These differences were felt to be due primarily to infections with rotavirus, an important cause of diarrhoea in children.

In a Canadian study comparing causes and rates of diarrhoea in northern and southern communities, rotavirus was also a key factor, with the rate of infection due to rotavirus in neonates significantly higher in the northern communities, especially in the far North (3). Infection due to another major viral pathogen, norovirus, was most common among the neonates of a northern community with a relatively unsafe water supply than in a southern community. The authors concluded that infection due to rotavirus appeared to be more frequent in the far North, whereas infection due to noroviruses appeared to be related more to inadequate sanitation.

Among the causes of bacterial gastroenteritis, outbreaks of enterohemorrhagic *E. coli* (EHEC) O157:H7 have been described in northern communities. This infection is of special concern since it can lead to hemolytic-uremic syndrome (HUS), as was seen in an outbreak in a Canadian Inuit community (4,5). Among parasitic infections, *Cryptosporidium* spp. and *Giardia lamblia* appear to be of potential importance in these communities, possibly due to consumption of food animals that carry these parasites (6).

Our understanding of the frequency and severity of gastrointestinal infectious agents in northern communities is poor, partially due to the limitations of currently used detection methods. For example, for viral detection, many laboratories in Canada still rely on electron microscopy, which has low sensitivity when compared to molecular methods, such as PCR. For bacterial infections, culture methods, the standard approach, are generally sensitive for detection of certain organisms, such as *Salmonella* spp., but may miss many other bacterial agents that do not grow well in culture. This is particularly the case when specimens are collected in remote communities necessitating long transit times, often under harsh conditions such as in the Canadian North. Culture is also a slow process, taking 2–4 days, so results are not available in time to help the health care worker with treatment decisions. Finally, for parasite detection, many laboratories use microscopic examination of stained stool specimens, with results not available for several days. Microscopic examination also appears to be much less sensitive than molecular methods for parasite detection (7,8). Given these problems, the cause of most diarrhoeal infections is not determined, even when stool samples are submitted for laboratory testing.

## Nanolitre PCR approach

Given the above limitations, innovative new tools and techniques for the rapid and comprehensive detection of food- and water-borne diarrhoeal agents are therefore urgently needed, especially in high-risk northern communities. We hypothesised that a technique known as nanolitre real-time PCR would be a useful method to investigate the prevalence of diarrhoeal pathogens in northern communities.

This technique involves a high-throughput quantitative real-time reverse transcriptase (RT)-PCR platform that enables over 3,000 separate PCR reactions to be performed in parallel in 33 nanolitre volumes in through-holes (analogous to wells on a microtitre plate). This reaction volume is approximately 1,000 fold less than is used in conventional single-assay PCR. Real-time RT-PCR assays (including the primers and 5′ exonuclease probe) for the desired targets are inserted in these through-holes by the manufacturer before being sent to the user. The extreme miniaturisation of volumes allows the user to perform multiple-target parallel PCR testing.

## Study aim

To use a nanolitre multiple-target real-time RT-PCR approach to investigate the prevalence of important bacterial, viral and parasitic causes of diarrhoea in a northern region, specifically the Qikiqtani (Baffin Island) Region of Nunavut, Canada.

## Main outcome measure

### Nanolitre PCR panel detection rate

Pathogen prevalence by nanolitre PCR calculated as:

- Number of specimens with at least 1 pathogen detected/number of specimens tested;
- Number of total pathogens detected/number of specimens tested; and
- Comparison of standard detection methods to the nanolitre PCR method for detection of targeted bacteria, parasites and viruses.

## Materials and methods

### Phase 1: Pathogen panel design

We first designed and evaluated an initial nanolitre PCR panel using specimens collected at the Children's Hospital of Eastern Ontario (CHEO, Ottawa, ON) laboratory that were reported to contain 1 or more of the target pathogens. Whenever possible, several different published PCR assays for each pathogen target were included in this prototype panel, so that they could be compared and the best one for each target could be selected for the second panel to be used with the study specimens from Nunavut.

This work was presented at the American Society of Microbiology, Emerging Technologies Meeting, San Juan, PR, March, 2011 (Appendix 1). The target assays referenced in Table I were those selected for inclusion in the panel used for this study.

**Table I:** Nanolitre real-time RT-PCR panel target pathogens

Bacteria	Viruses	Parasites
Enterohemorrhagic *Escherichia coli* (EHEC)	Norovirus group 1 (20)	*Giardia lamblia* (25)
*stx* I^a^ (9)		
*stx* II^a^ (10)		
*E. coli* O157 (11)		
*Salmonella* spp. (12)	Norovirus group 2 (20)	*Cryptosporidium* spp. (26)
*Shigella* spp. (13)	Rotaviruses (21)	
*Campylobacter* spp. (14)	Astroviruses (22)	
*Yersinia entercolitica* (15)	Adenoviruses 40/41 (23)	
*Clostridium difficile* (16)	Sapoviruses (24)	
*Clostridium difficile tcd* B^b^ (17)		
*Listeria monocytogenes* (18)		
*Vibrio parahaemolyticus* (19)		

Number in brackets indicates number in reference list for publication describing assay.

a *stx* I and *stx* II are genes encoding for Shiga toxin 1 and 2, one of which is generally present in all strains of EHEC.

b *tcd* B is the gene encoding for *C. difficile* toxin B, the key virulence factor.

### Phase 2: Evaluation of panel using diarrhoeal stool specimens from Nunavut

In the second phase, we tested diarrhoeal faecal samples submitted to the Qikiqtani General Hospital Laboratory in Iqaluit, Nunavut, with the second nanolitre panel. We also compared conventional and nanolitre PCR test results for these specimens.

Qikiqtani General Hospital (QGH) serves the Qikiqtani (Baffin Island) Region of Nunavut. Stool samples were only submitted from outpatient clinics in communities in this region as well as patients seen at QGH.

Ethics approval for this study was obtained from both the CHEO Research Ethics Committee and Nunavut Research Institute. Given that this was a prospective study and consent was not obtained from individual subjects, the only information collected was date of stool submission and results of conventional microbiology testing on individual stool samples. Samples were otherwise made anonymous to the investigators.

#### Specimen collection and testing

All diarrhoeal stool samples submitted to QGH laboratory for bacterial culture over an 18 month period (January 2010 until June 2011) were sent as per routine practice to an accredited clinical laboratory in Edmonton, Alberta, Canada, for testing following standard culture procedures. Some samples were also sent for microscopic and enzyme immunoassay (EIA) examination for parasites, electron microscopy for viruses, and/or *Clostridium difficile* toxin testing as per the clinician request. Residual volumes of these samples that would otherwise be discarded were saved at −20°C and shipped frozen monthly to the CHEO research laboratory in Ottawa, Ontario, Canada, for nanolitre PCR testing.

#### Nucleic acids extraction

Nucleic acids were extracted from faecal specimens using 2 commercial methods. The Zymo faecal DNA kit (Zymo Research, Irvine, CA) was used in order to extract DNA for bacteria and parasites, as this method included a bead-beating step designed to lyse organisms with thick cell walls. This method was performed following the manufacturer's recommendations. The PureLink Viral extraction kit (Life Technologies Corp., Carlsbad, CA) was used for extraction of viral nucleic acid. This method was performed on an automated extraction device (iPrep, Life Technologies Corp.). Based on preliminary experiments, the nucleic acids obtained with this method were additionally filtered to remove PCR inhibitory substances in the stool using the Zymo-Spin IV HRC Spin Filter (Zymo Research).

#### Targets and PCR assays

Based on our findings from this CHEO study, we selected the best assays for inclusion in a second PCR panel for use with the Nunavut specimens. The target pathogens are shown in Appendix 1.

#### Controls

Nucleic acid extraction and RT-PCR amplification was monitored using appropriate controls. Amplification inhibition is of special concern when working with stool, since inhibitory substances may be present. We used 2 microorganisms as extraction and amplification controls. These were MS2 bacteriophage (Zeptometrix Corp., Buffalo, NY) and the bacterium *Bacillus atrophaeus* [American Type Culture Collection (ATCC), Manassas, VA]. Both controls were added to stool specimens prior to nucleic acid extraction.

#### PCR assays

PCR assays used were 5′ exonuclease probe assays that had been previously published and validated. These were purchased from Idaho Technologies (Coralville, ID).

#### Nanolitre PCR

A 1-step real-time PCR protocol was used following manufacturer's directions (OpenArray, Life Technologies Corp., Carlsbad, CA). Briefly, selected PCR assays (primers and probe) were inserted in duplicate in through-holes of each subarray on the OpenArray plates (i.e. each OpenArray plate has 48 through-holes, squares or subarrays of 64 through-holes each) by the manufacturer and shipped to our laboratory. Master mix was prepared using Super Script III Platinum One Step qRT-PCR System (Invitrogen, Grand Island, NY) and aliquoted into a 12 by 4 rectangular area of a 384-well MatriPlate corresponding to 1 OpenArray plate. Sample nucleic acid was then added to its corresponding well of the 384-well plate. Nucleic acid extracts were tested in duplicate (2 subarrays for each extract, or 4 through-holes per extract in total).

A sample tip block containing 48 pipette tips was placed above the 48 wells on the 384-well plate and the nucleic acid sample-master mix mixture was loaded into the pipette tips by manually sliding up and down the tip block. The tip block and the OpenArray plate were then placed into an auto-loader device where the sample from each pipette tip was distributed into the through-holes of 1 subarray. The nucleic acid sample-master mix mixture was moved into the through-holes by hydrophilic forces. The loaded OpenArray plate was then inserted into a glass slide case filled with an immersion fluid provided by the manufacturer. The top of the glass case was sealed with UV glue that was then cured with a UV light device provided with the thermocycler. This resulted in a fully closed PCR amplification chamber.

#### Thermocycling

The plate was then placed in the OpenArray thermocycler and a customised 1-step thermocycling protocol was begun. This consisted of an initial reverse transcription phase followed by 40 amplification cycles, taking approximately 2 hours to complete. The crossing threshold values were recorded and analysed.

#### PCR interpretation

Based on manufacturer's recommendations, samples were considered positive if the cycle threshold was less than 30 on the nanolitre PCR assay. The nucleic acid extracted with the Zymo method was used for interpretation of PCR results for parasites and bacteria, and the nucleic acid extracted with the iPrep Pure Link Viral method was used for interpretation of viral PCR results.

## Results

### Nunavut PCR study

The nanolitre real-time RT-PCR results for the diarrhoeal stool samples are shown in Table II. Eighty-six stool specimens were submitted for bacterial culture, and all of these were studied with the PCR panel. Of these specimens, 20 had also been submitted for parasite examination [18 by microscopy, 2 by enzyme immunoassay (EIA) for *Cryptosporidium* spp. and *Giardia lamblia*], and 2 samples had undergone viral testing by electron microscopy. None of these specimens had been sent for *C. difficile* testing by clinicians. Among the 86 stool specimens tested by PCR, a total of 50 pathogens were detected with 1 or more pathogens found present in 40 (46.5%) stool specimens. The organisms detected comprised 17 *Cryptosporidium* spp., 11 *C. difficile* including 5 with toxin B, 6 *Campylobacter* spp., 6 *Salmonella* spp., 4 astroviruses, 3 noroviruses, 1 rotavirus, 1 *Shigella* spp. and 1 *Giardia* spp.

**Table II:** Nanolitre real-time RT-PCR panel results on the detection of food- and water-borne microbial agents in northern communities

Microorganism	Nanolitre PCR positives (N=86) (%)
Bacteria	
* Campylobacter* spp.	6 (7.0)
* Salmonella* spp.	6 (7.0)
* Clostridium difficile* with toxin B detected	5 (5.8)
* Shigella* spp.	1 (1.1)
Parasites	
* Cryptosporidium* spp.	17 (19.8)
* Giardia* spp.	1 (1.1)
Viruses	
* *Astroviruses	4 (4.6)
* *Noroviruses groups 2	3 (3.5)
* *Rotaviruses	1 (1.1)

Thirty-two of the 40 specimens in which a pathogen was detected (80%) contained a single organism. In 8 specimens (20%), more than 1 pathogen was detected, 6 contained 2 pathogens (*Campyloba*cter spp./*Cryptosporidium* spp.; *Cryptosporidium* spp./*C. difficile*; norovirus/*C. difficile; Salmonella* spp./*C. difficile* and *C. difficile* toxins; *Salmonella* spp./astrovirus; and astrovirus/*C. difficile* and *C. difficile* toxins), whereas in the other 2 specimens 3 pathogens were detected (*Campylobacter* spp./norovirus/*C. difficile*; *Shigella* spp./astrovirus/*Campylobacter* spp.).

Conventional method results showed that a bacterial pathogen was reported by culture from 10/86 samples. These comprised 7 *Salmonella* spp., 1 *Campylobacter* spp., 1 *Aeromonas* spp. and 1 *Vibrio parahaemolyticus*. No pathogens were reported in 19/20 specimens tested for parasites. The parasite *Blastocystis hominis* was reported in 1/20 specimens. The 2 samples in which electron microscopy was used for viral detection were both reported as negative.

Comparing PCR results to the standard tests for *Salmonella* spp. detection, indicated that 2 samples (both from a single patient) were culture-positive but PCR-negative, and 1 sample was PCR-positive but culture-negative. For *Campylobacter* spp., the single culture-positive specimen was also PCR-positive for the same organism. Five additional samples were *Campylobacter* PCR-positive but culture-negative. *Aeromonas* spp. assay was not included in the PCR panel as *Aeromonas* spp. status as a pathogenic agent is uncertain. By PCR, the sample that contained *Aeromonas* spp. by culture was positive for *C. difficile* toxin and for astrovirus. *V. parahaemolyticus* was included in the nanolitre PCR panel but the toxin gene assay for this organism was negative in the culture-positive sample. This sample was therefore studied further using microlitre volume PCR assays. A *Vibrio* spp. assay was positive but assays for the 2 major virulence genes that enable this organism to cause diarrhoea were both negative by PCR, indicating this organism isolated in culture may have been a non-virulent strain.

With respect to parasite detection, 5 of the 18 PCR-positive specimens had undergone testing by microscopy or EIA, and no parasites were detected in these samples. The 2 samples negative for viruses by electron microscopy were also negative by PCR.

## Discussion

Our finding that *Campylobacter* spp. was detected more often by PCR than culture has also been reported by others (27). Prolonged transportation time may have impaired the ability of these organisms to grow in culture, as environmental conditions may cause *Campylobacter* to enter what is known as a viable but non-culturable state (VBNC). Even in a setting where prolonged transportation time was not a factor, a recent report on a molecular detection method compared to culture found that PCR still detected *Campylobacter* spp. much more frequently than culture. Among PCR-positive specimens, 46.8% did not grow in culture (28). Approximately half of these were found through the use of additional PCR and sequencing methods to be common species of *Campylobacter* spp. such as *C. jejuni*, while other PCR-positive/culture-negative specimens contained other *Campylobacter* species that cannot be grown using standard methods.

With respect to the routes of transmission of *Campylobacter* spp. in Nunavut, these bacteria are generally considered food-borne pathogens, but person-to-person and zoonotic (animal-to-human) transmission routes are also possible. Of interest, various *Campylobacter* species were detected by PCR and sequencing in 75% of faecal specimens from dogs in a northern community in Canada (29). The most prevalent species were *C. jejuni* and *C. upsaliensis*, each found in 33% of dogs. The authors of this study concluded that dogs may be a source of human infections in such settings where canine carriage is common.

Additional investigations into the discrepancy in PCR and culture results for *Campylobacter* detection, which we observed in our study, are needed. By performing species-specific PCR and sequencing, we hope to determine which species of *Campylobacter* are present in the PCR-positive/culture-negative samples. This information should help establish the source or sources of this infection in Nunavut.

The detection of the parasite *Cryptosporidium* spp. by PCR in a relatively large number of specimens was also striking. *Cryptosporidium* infections in humans are most commonly caused by 2 species, *C. parvum* and *C hominis*. However, the PCR assay used was a broad-spectrum assay designed to detect many different species including animal pathogens. We specifically chose to use this assay in the panel as we wished to see if any uncommon species were present in Nunavut residents due to environmental and dietary exposures that differ from those in southern Canada. Also of note, in other parts of the world where under-nutrition/malnutrition is also a concern *Cryptosporidium* spp. infection has been associated with poor growth and development in children (30,31).

As with *Campylobacter*, we now plan to perform additional molecular studies to determine which *Cryptosporidium* spp. are present in the stool samples which may provide an indication of the sources of infection. *Cryptosporidium* spp. can be transmitted in several ways including through water, the environment, and person-to-person. In the North, *Cryptosporidium* spp. has been described in ringed seals and bearded seals in Nunavik, QC, a region that borders Nunavut, making food-borne transmission through the consumption of seals or other marine mammals a possible route (6). Dogs have also been found to carry *Cryptosporidium* in northern Canada (32) and may be another source of zoonotic infection.

As with *Campylobacter* spp., a discrepancy between the results of standard methods and PCR was seen for *Cryptosporidium* spp. The reasons for this are uncertain, but again as with *Campylobacter*, molecular detection rates appear to be higher than detection by microscopic methods (7,8,33). It is possible that the concentration of *Cryptosporidium* spp. in the samples was below the threshold at which organisms can be detected by microscopy, but could still be detected by PCR due to the higher sensitivity of this method.

*Clostridium difficile*, and the pathogenic toxin B of this organism, were also detected in a substantial proportion of specimens. *C. difficile* toxin B gene detection, as seen in 5 stool samples, is generally accepted as evidence that the diarrhoeal episode is due to *C. difficile*. The detection of the organism alone without the presence of toxin B (as in 6 samples in this study) may reflect asymptomatic carriage. Such carriage is common in infants, but due to the study design we were unable to determine the age of the patients.

Future work with respect to specimens positive for *C. difficile* will be performed to determine if the strain of *C. difficile* originally described from Quebec, that is, associated with severe disease and high mortality is present in any of the Nunavut samples.

The finding of a PCR-positive sample for *Shigella* spp. that was culture-negative is also of interest. This PCR assay uses a virulence gene target common to both *Shigella* spp. and a strain of *E. coli* known as enteroinvasive *E. coli* (EIAC). Since EIAC is not known to be present in Canada, our assumption is that *Shigella* was present in this sample, but this will also be confirmed by additional testing.

The absence of detection of certain pathogens is also notable. For example, no shigatoxin-producing *E. coli* were detected by culture or PCR methods, despite outbreaks of this pathogen in the North in the past. *Listeria monocytogenes* was also not detected by PCR; *L. monocytogenes* is considered a rare cause of diarrhoeal disease but is notable for its ability to cause life-threatening illness in immunocompromised patients, as well as in infants and the elderly, as seen in a large Canadian outbreak in recent years (34).

Despite being reported as a common pathogen in earlier studies on infants and children in the North, rotavirus was detected in only 1 specimen by PCR. Norovirus was also detected relatively infrequently. However, this may have been due to the study design, as only samples submitted by clinicians for bacterial culture were included in the study, which may have selected for more persistent or severe inflammatory-type diarrhoeal infections, as opposed to viral gastroenteritis which in general is shorter in duration and non-inflammatory. Furthermore, rotavirus is generally a significant pathogen mainly in young children, while our study examined specimens from patients of all ages.

There are several limitations of the study that should be mentioned. A main limitation was that clinical and demographic information was not collected as this was a laboratory based prospective study and individual consent was not obtained. It is therefore not possible to determine the age or geographic distribution of these diarrhoeal cases. We also passively collected aliquots of specimens submitted to the lab for testing (predominantly for bacterial culture), and this may therefore represent a biased sample of either more severe or persistent cases of diarrhoea. Also as with all molecular tests, it can be argued that the presence of nucleic acids for an organism does not verify the actual presence or viability of that organism, and potentially could reflect a past infection with that organism with persistence of nucleic acids. PCR detection of certain organisms in the faeces may also be due to viable (live) organisms that are colonising the patient but are not necessarily the cause of the diarrhoeal episode. For example, *C. difficile* can be carried by young infants and not lead to diarrhoea. Similarly, *Cryptosporidium* spp. and *Giardia lamblia* can also be detected in some patients who do not have gastrointestinal symptoms. Thus, we cannot assume that these parasites were responsible for the diarrhoeal infection in the patients from whom they were detected. Finally, although 1 or more pathogenic organisms were detected in almost half the specimens tested, further investigations are needed to look for additional infectious agents that were not included in the PCR panel used in this study.

In summary, our results are notable in that the importance of 3 infectious agents may be under-recognised in Nunavut residents with diarrhoea. Further study is needed to determine the epidemiology and clinical significance of these organisms in this region.

We also conclude that nanolitre real-time RT-PCR testing for multiple infectious agents simultaneously is a useful tool for gastrointestinal pathogen research and potentially may also be helpful for clinical diagnostics and outbreak investigation in remote regions where molecular methods may have advantages over currently used non-molecular methods. The nanolitre real-time RT-PCR technique revealed the presence of a number of pathogens that were either not detected by standard testing or were found in samples for which standard testing for the pathogen was not performed. Most notably, results suggest that *Cryptosporidium* spp., *Campylobacter* spp. and *C. difficile* may be relatively common but possibly under-recognised pathogens in Qikiqtani region residents of Nunavut, Canada.

## Appendix 1. Phase 1 Results

**ASM Emerging Technologies abstract**

*Author Block:* R. Slinger^1^, D. Goldfarb^2^, G. German^3^, N. Barrowman^1^, I. Moldovan^2^, F. Chan^1^; ^1^University of Ottawa, Children's Hospital of Eastern Ontario, Ottawa, ON, Canada, ^2^Children's Hospital of Eastern Ontario, Ottawa, ON, Canada, ^3^University of Ottawa, Ottawa, ON, Canada.

Abstract

**Background:** Diarrhoea can be caused by many different bacteria, viruses, and parasites. The methods currently used to detect these agents have major limitations. For example, the methods are capable of detecting only 1 type of agent, when any 1 of many agents may be responsible; results from culture methods require several days; and non-culture methods such as EIA suffer from both false positive and false negative results. We sought to make detection of diarrhoea-causing infectious agents more rapid (3.5 hours) and simpler by using a nanolitre real-time PCR panel capable of simultaneous detection of multiple infectious agents.

**Methods:** We designed a multi-target nanolitre real-time PCR platform (OpenArray™ system, Life Technologies) containing previously validated 5′ exonuclease PCR assays for major diarrhoeal pathogens including bacteria (*Clostridium. difficile, Salmonella* spp., *Campylobacter* spp., *Escherichia coli* 0157:H7, *Shigella* spp., and *Yersinia enterocolitica*), viruses (rotaviruses, noroviruses, and astroviruses); and parasites (*Giardia* spp., *Cryptosporidium* spp., and *Dientamoeba fragilis*). We evaluated this nanolitre PCR panel using a collection of saved faecal samples that had tested positive with conventional tests.

**Results:** The nanolitre PCR Diarrhoea panel detected the pathogen identified by conventional methods in 66/68 specimens (97.1%; 95% CI 89.9%–99.2%). This included 17/18 *Campylobacter* spp., 15/16 *Salmonella* spp., 9/9 *C. difficile*, 5/5 *E. coli* 0157:H7, 1/1 *Shigella* spp., and 1/1 *Y. enterocolitica*, 9/9 rotaviruses, 3/3 noroviruses, 2/2 astroviruses, 1/1 *Giardia* spp., 2/2 *Cryptosporidium* spp., and 1/1 *D. fragilis*.

**Conclusions:** Sensitive simultaneous detection of important bacterial, viral, and parasite diarrhoeal pathogens using validated 5′ exonuclease assays was possible on the OpenArray™ nanolitre real-time PCR platform. The simplicity and the speed of this approach (approximately 3.5 hours, including nucleic acid extraction) are major improvements relative to current laboratory detection methods for diarrhoeal pathogens. We plan to perform a prospective evaluation of the panel, with the aim of developing a comprehensive single assay for detection of diarrhoeal agents.

**Table:** Results table

Infectious agent	Standard tests: number detected	Nonolitre PCR panel: number detected
*Campylobacter* spp.	18	17
*Salmonella* spp.	16	15
*Clostridium difficile*	9	9
*E. coli* 0157:H7	5	5
*Shigella* spp.	1	1
Yersinia enterocolitica	1	1
Rotavirus	9	9
Noroviruses	3	3
Astroviruses	2	2
*Giardia* spp.	1	1
*Cryptosporidium* spp.	2	2
*D. fragilis*	1	1
Total	68	66
